# Circular RNA *circSVIL* Promotes Myoblast Proliferation and Differentiation by Sponging miR-203 in Chicken

**DOI:** 10.3389/fgene.2018.00172

**Published:** 2018-05-16

**Authors:** Hongjia Ouyang, Xiaolan Chen, Weimin Li, Zhenhui Li, Qinghua Nie, Xiquan Zhang

**Affiliations:** ^1^Department of Animal Genetics, Breeding and Reproduction, College of Animal Science, South China Agricultural University, Guangzhou, China; ^2^College of Animal Science and Technology, Zhongkai University of Agriculture and Engineering, Guangzhou, China; ^3^National-Local Joint Engineering Research Center for Livestock Breeding, Guangdong Provincial Key Lab of Agro-Animal Genomics and Molecular Breeding, and the Key Lab of Chicken Genetics, Breeding and Reproduction, Ministry of Agriculture, Guangzhou, China

**Keywords:** circular RNA, *circSVIL*, miRNA, miR-203, skeletal muscle, proliferation, differenation

## Abstract

Circular RNAs (circRNAs), expressed abundantly and universally in various eukaryotes, are involved in growth and development of animals. Our previous study on circRNA sequencing revealed that *circSVIL*, an exonic circular, expressed differentially among skeletal muscle at 11 embryo age (E11), 16 embryo age (E16), and 1 day post-hatch (P1). In this study, we aim to investigate the effect of *circSVIL* on the development of skeletal muscle. We detected the expression level of *circSVIL* in embryonic leg muscle during E10 to P1. As a result, we found that *circSVIL* had a high expression level during late embryonic development of skeletal muscle. Through dual-luciferase assay, RNA immunoprecipitation and biotin-coupled miRNA pull down, we found chicken *circSVIL* could functions as miR-203 sponges and upregulated the mRNA level of *c-JUN* and *MEF2C*. In chicken, *circSVIL* could promote the proliferation and differentiation of myoblast, and antagonize the functions of miR-203. Altogether our data suggest that *circSVIL* promotes the embryonic skeletal muscle development by sequestering miR-203 in chicken.

## Introduction

Skeletal muscle is the most important component of body, and moreover, it is directly correlated with meat production in domestic animals (Güller and Russell, 2010). The growth and development of skeletal muscle is controlled by a very complex biological process, which could be regulated by many genes, transcription factors, as well as some non-coding RNAs (Buckingham et al., 2003; Bassel-Duby and Olson, 2006; van Rooij et al., 2008; Neguembor et al., 2014). MicroRNAs (miRNAs) have been extensively studied and characterized as crucial components within the regulatory network for muscle development. Several well-known myogenic miRNAs, such as miR-1, miR-206, and miR-133, were reported to regulate muscle development by inhibiting target gene expression (Anderson et al., 2006; Chen et al., 2006). MiR-203 was well-known as a tumor suppressor, but it was found also involving in muscle development recently (Furuta et al., 2010). MiR-203 negatively regulates the proliferation of smooth muscle cells, and was detected abundant expressed in C2C12 myoblasts, quail myoblasts and chicken skeletal muscle (Chen et al., 2006; Li et al., 2011; Liao et al., 2015). In zebrafish embryos, miR-203 regulates fast muscle differentiation by targeting dmrt2a (Lu et al., 2017).

CircRNAs, as a novel class of endogenous RNAs, have been identified from various cell types or tissues of eukaryote in recent years (Salzman et al., 2013; Wang et al., 2014; Westholm et al., 2014; Ye et al., 2015). Abundantly expressed in skeletal muscle, some circRNAs were found to be associated with muscle growth and development in monkey, pig and chicken (Abdelmohsen et al., 2015; Liang G. et al., 2017; Ouyang et al., 2017). Although the biological roles of circRNAs have not been clearly understood, some circRNAs could act as miRNA sponges and are therefore related to many diseases such as cancer (Yamamura et al., 2017). The most representative example is that circRNA *CDR1as*, also known as *ciRS-7*, affects brain function in zebrafish and mouse by sponging miR-7 efficiently (Hansen et al., 2013; Memczak et al., 2013; Piwecka et al., 2017). In previous study, we found two circRNAs produced by the RBFOX2 gene promoted the proliferation of muscle cells by binding with miR-1a-3p and miR-206 (Ouyang et al., 2017). In addition, several circRNAs have also been demonstrated to be associated with cancers by sponging miRNAs (Huang R et al., 2017; Huang X. Y et al., 2017; Jin et al., 2017; Liang H. F. et al., 2017).

A circRNA generated from exon 6 to 14 of supervillin (SVIL) gene (*circSVIL*), is the most differentially expressed circRNA among three developmental stages of muscle in chickens, as identified by our previous study on circRNAs sequencing (Ouyang et al., 2017). We predicted that it has four potential binding sites for miR-203, which has been shown to inhibit skeletal muscle proliferation and differentiation by repressing *c-JUN* and *MEF2C* (Luo et al., 2014). In this study, we aim to validate the interaction between *circSVIL* and miR-203, and further to investigate the effect of *circSVIL* on myoblast proliferation and differentiation.

## Materials and methods

### Ethics standards

Animal experiments were approved by the Animal Care Committee of South China Agricultural University (Guangzhou, China) with approval number SCAU#0014. All experiments were handled in compliance and all efforts were made to minimize suffering.

### Animals and cells

A total of 240 Xinghua chickens at 10-day embryonic age (E10) were obtained from the Chicken Breeding Farm of South China Agricultural University (Guangzhou, China), and incubated in Automatic Incubator (Oscilla, Shandong, China) at 37.8°C, with 60 ± 10% humidity. Leg muscles of 20 Xinghua chickens were collected every day from E10 to 1 day post-hatch (P1).

Chicken primary myoblasts were isolated from the leg muscles of E11 chickens as described by our previous study (Ouyang et al., 2017). Primary myoblasts were cultured in Dulbecco's modified Eagle medium (DMEM) (Gibco) with 20% fetal bovine serum (Gibco) and 0.2% penicillin/streptomycin (Invitrogen, Carlsbad, CA, USA) at 37°C in a 5% CO_2_, humidified atmosphere. DF-1 cells were also cultured in DMEM but with 10% fetal bovine serum. QM-7 cells were cultured in high-glucose M199 medium (Gibco) with 10% fetal bovine serum, 10% tryptose phosphate broth solution (Sigma, Louis, MO, USA), and 0.2% penicillin/streptomycin.

### Total RNA isolation, cDNA synthesis, and quantitative real-time PCR (qRT-PCR)

Total RNAs were isolated using Trizol Reagent (Invitrogen), following the manufacturer's instructions. The synthesis of cDNA was performed using RevertAid First Strand cDNA Synthesis Kit (Fermentas, Waltham, MA, USA) with random hexamers. The qRT-PCR was performed using SsoFast Eva Green Supermix (Bio-Rad, Hercules, CA, USA) in a BIO-RAD CFX96 system as follows: 95°C for 3 min; 40 cycles of 95°C for 10 s, annealing temperature (58–62°C) for 30 s, and 72°C for 30 s; and a final extension at 72°C for 1 min. The relative expression level of gene was calculated using the comparative 2^−ΔCt^ (ΔCt = Ct_target gene_ – Ct_reference gene_). Fold change values were calculated using the comparative 2^−ΔΔCt^, in which ΔΔCt = ΔCt (target sample) – ΔCt (control sample). All reactions were run in triplicate and presented as means ± S.E.M. The Student's *t-test* was used to compare expression levels among different groups.

Primers used for qRT-PCR were designed using Premier Primer 5.0 software (Premier Bio-soft International, Palo Alto, CA, USA) and synthesized by Biosune Co. Ltd (Shanghai, China). Primers used for circSVIL was designed as divergent primers to detect backsplice junctions (Jeck et al., 2013), while for linear SVIL was normal convergent primers. The *GAPDH* and *18S rRNA* gene were used as reference genes. Details of primers were summarized in Table S1.

### Vector construction, RNA oligonucleotides, and cell transfection

The wild type and mutated sequences of *circSVIL* (harbored normal and mutated miR-203 perfected 5′ seed pairing binding site) were synthesized and cloned into the pmirGLO dual-luciferase reporter vector (Promega) using the *NheI* and *XhoI* restriction sites. The perfect match sequence of gga-miR-203 was synthesized and cloned into the psiCHECK-2 vector (Promega) using the *NotI* and *XhoI* restriction sites. The overexpression vectors of *circSVIL* were constructed by using pCD-ciR2.1 vector (Geneseed Biotech, Guangzhou, China). The linear sequence of *circSVIL* was synthesized and cloned into pCD-ciR2.1 according to the manufacturer's protocol using the *KpnI* and *BamHI* restriction sites. Small interfering RNA (siRNA) of *circSVIL* (sequences are summarized in Table S1) and miRNA mimics were synthesized by Ribobio Co. Ltd (Guangzhou, China).

Cells were transfected with 50 nM of miRNA mimics, 100 nM of siRNA or plasmid (1 μg/mL) using Lipofectamine 3000 reagent (Invitrogen) according to the manufacturer's instructions.

### Luciferase reporter assay

For pmirGLO vector, DF-1 cells were seeded in 96-well plates and co-transfected with wild type (WT) or mutated reporter vector and miR-203 mimics or NC (negative control). For psiCHECK-2 vector, DF-1 cells were co-transfected with reporter vector and miR-203 mimics or NC and with *circSVIL* overexpression vector or empty vector (EV).

After transfected for 48 h, the luminescent values of *firefly* and *Renilla* luciferase were detected using Dual-GLO Luciferase Assay System Kit (Promega) with a Fluorescence/ Multi-Detection Microplate Reader (Biotek, Winooski, VT, USA).

### RNA immunoprecipitation and biotin-coupled miRNA pull down

Overexpression vector of *circSVIL* was transfected into QM7 cells along with miR-203, miR-206, or miR-NC control. After transfected for 48 h, Immunoprecipitation of AGO2 was performed using the Magna RIP RNA-Binding Protein Immunoprecipitation Kit (Millipore, Bedford, MA) following the manufacturer's instructions. The mRNA levels of *circSVIL* were quantified by qRT–PCR and were normalized to *GAPDH* gene. The relative immunoprecipitate/ input ratios are plotted.

QM7 cells were co-transfected with *circSVIL* overexpression vector and 3′ end biotinylated miR-203, miR-206, or miR-NC (RiboBio). After transfected for 48 h, the biotin-coupled RNA complex was pull-downed by Dynabeads MyOne Streptavidin C1 kit (Invitrogen) following the manufacturer's instructions. Then the RNAs bound to the beads (pull-down RNA) were isolated using Trizol LS reagent (Invitrogen). The mRNA levels of *circSVIL* in the streptavidin captured fractions were quantified by qRT–PCR and the relative enrichment ratios were plotted.

### Flow cytometry analysis of the cell cycle

After overexpression or knockdown the *circSVIL*, the transfected myoblasts were collected and fixed in 70% ethanol overnight at −20°C. Then, the cells were collected and incubated with 50 μg/ml propidium iodide (Sigma), 10 μg/mL RNaseA (Takara), and 0.2% (v/v) Triton X-100 (Sigma) for 30 min at 4°C. Analyses were performed using a BD AccuriC6 flow cytometer (BD Biosciences, San Jose, CA, USA) and FlowJo (v7.6) software (Treestar Incorporated, Ashland, OR, USA).

### EdU assay

After overexpression or knockdown the *circSVIL*, the proliferation of myoblasts was tested using a Cell-Light EdU Apollo 567 *in vitro* Flow Cytometry Kit (Ribobio). The cells were exposed to 50 μM EdU for 2 h at 37°C following the manufacturer's instructions.

The EdU-stained cells were visualized by fluorescence microscopy (Nikon, Tokyo, Japan). The analysis of cell proliferation was performed using images of randomly selected fields obtained from the fluorescence microscope. We performed four repeats for each group, and three images were used to calculate the cell proliferation rate in each repeats.

### Western blotting

Proteins of transfected myoblasts were extracted using lysis buffer and the concentration determined by a bicinchoninic acid (BCA) protein assay kit (Beyotime, Shanghai, China). Total proteins (50 μg) were separated on a 12% SDS-PAGE, and transferred to a polyvinylidene difluoride membrane (Millipore, Bedford, MA). After blocked in 5% BSA blocking solution for 1 h at room temperature, the membrane was incubated overnight at 4°C with primary antibodies against MYOG (Biorbyt, Cambridge, UK; diluted 1:500) and MHC (DSHB, Iowa, USA; diluted 1:1,000). Subsequently, the membrane was developed with anti-rabbit or anti-mouse horseradish peroxidase conjugated secondary antibodies (Sigma-Aldrich, diluted 1:5,000). Protein bands were visualized using enhanced chemiluminescence (ECL) system (GE Healthcare, USA) and quantified with an ImageQuant LAS4000 system (Fujifilm, Tokyo, Japan).

## Results

### Circular RNA circSVIL differentially expressed during chicken embryonic leg muscle development

**Figure 1:**
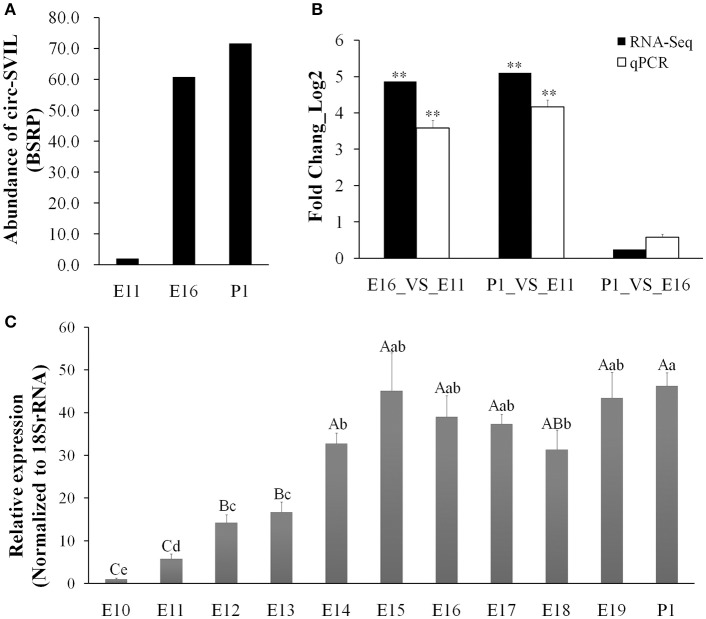
Chicken *circSVIL* is abundant and dynamically expressed during embryonic leg muscle development. **(A)** The RNA-Seq result showed that *circSVIL* differentially expressed in E11, E16, and P1 of leg muscle. The expressed abundances of circRNAs were normalized as number of back-spliced reads per million mapped reads (BSRP). **(B)** qRT-PCR validation of *circSVIL* in E11_VS_E16, E11_VS_P1 and E16_VS_P1 comparisons. **(C)** The expression of *circSVIL* in leg muscle during E10 to P1 was detected by qPCR. The Student's *t*-test was used to compare expression levels among different groups. ^*^P < 0.05; ^**^P < 0.01; ^a,b^*P* < 0.05; ^A,B^*P* < 0.01.

Our previous circRNAs sequencing date (available in the Gene Expression Omnibus with accession number GSE89355) found that some circRNAs abundant and differentially expressed during embryonic leg muscle development in chicken. The *circSVIL* was the most abundant and differentially expressed exonic circRNA as discovered by our previous sequencing results (Ouyang et al., 2017). The expression of *circSVIL* was significantly higher in the leg muscle of E16 and P1 than that in E11 (Figure 1A). The *circSVIL* was validated by divergent reverse-transcription PCR and RNaseR digestion in our previous studies (Figure S1) (Ouyang et al., 2017). The expression level of *circSVIL* at E11, E16 and P1 was also confirmed by qRT-PCR. The expression patterns of *circSVIL* in E16_VS_E11, P1_VS_ E11, and P1_VS_ E16 comparison groups were consistent with the RNA-Seq results (Figure 1B). Moreover, we detected the expression of *circSVIL* in chicken embryonic leg muscle during E10 to P1. The results showed that the expression of *circSVIL* increased sharply during E11–E14, and then maintained at a high expression level from E14 to P1 (Figure 1C).

### CircSVIL interacts with miR-203 in myoblasts

**Figure 2:**
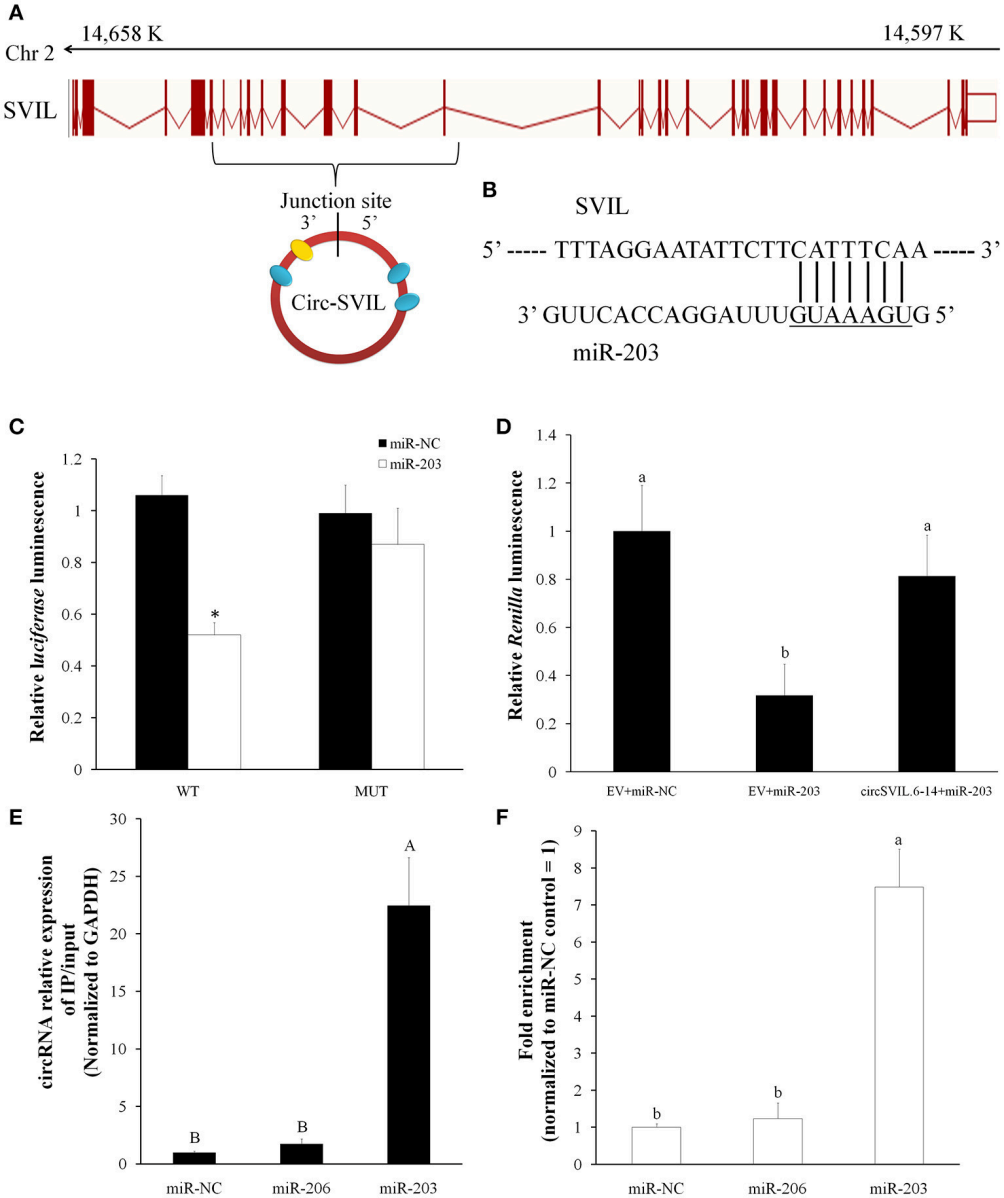
Chicken *circSVIL* interacted with miR-203. **(A)** Chicken *circSVIL* is drive from exon 6 to 14 of *SVIL* gene and harbored four miR-203 binding sites. The yellow oval indicates perfected 5′ seed pairing binding site of miR-203, and blue ovals indicate other three potential binding sites of miR-203. **(B)** The perfected 5′ seed pairing binding sites of miR-203 in *circSVIL*. Mut indicates the mutation sequences of binding sites. **(C)** DF-1 cells were co-transfected with wild-type (WT) or mutant (MUT) luciferase reporters with miR-203 mimic or control duplexes. The relative levels of *firefly* luminescence normalized to *Renilla* luminescence are plotted. Error bars represent S.D. (*n* = 6). **(D)** Luminescence was measured 48 h after co-transfected with the luciferase reporter and miRNA mimics or negative control (NC) and with circRNAs overexpression vector or empty vector (EV). The relative levels of *Renilla* luminescence normalized to firefly luminescence are plotted. Error bars represent S.D. (*n* = 6). **(E)** Immunoprecipitation of AGO2 from myoblasts co-transfected with miR-203, miR-206 or miR-NC, and *circSVIL* overexpression vector. The expression level of *circSVIL* was quantified by qRT–PCR and normalized by GAPDH, and the fold change of immunoprecipitate/input are plotted. Error bars represent S.D. (*n* = 3). **(F)** RNA pull-down from the myoblast lysates after transfection with 3′ end biotinylated miR-1a, miR-206, or miR-203 control. The expression level of *circSVIL* was quantified by qRT–PCR, and fold enrichment in the streptavid in captured fractions are plotted. Error bars represent S.D. (*n* = 3). Student's *t*-test (two-tailed) was performed for data analysis. ^*^P < 0.05; ^**^P < 0.01; ^a,b^*P* < 0.05; ^A,B^*P* < 0.01.

We identified the sequence of circular RNA *circSVIL*, and found it was derived from exon 6 to 14 of *SVIL* (ENSGALT00000011863.4) and located within 14,597,995-14,657,468 region (Gallus_gallus-4.0/galGal4) at the reverse strand of chromosome 2 (Figure 2A). After further analysis of its sequence using miRanda, *circSVIL* was predicted to possess a miR-203 perfected 5′ seed pairing binding sites at 1303-1309 nt (Figures 2A,B). Three other potential binding sites for miR-203 in *circSVIL* were also predicted using RNAhybrid (Figure 2A and Figure S2).

To validate the relation between *circSVIL* and miR-203, we constructed a dual-luciferase reporter (pmirGLO) by fused the wild-type (WT), or mutant (MUT) linear sequence of *circSVIL* (Figure 2B) in the 3′ end of *firefly* luciferase and performed luciferase reporter assay in DF-1 cells. Compared to miR-NC, the *firefly* luciferase activity of the WT plasmid was significantly reduced by miR-203 (*P* < 0.05), whereas the mutant reporter has no response to miR-203 (Figure 2C). We used the other dual-luciferase reporter (psiCHECK-2) by inserting perfect miR-203 target sequence into the 3′ end of *Renilla* luciferase. After co-transfected this dual-luciferase reporter with miR-203 in DF-1, we found the relative luminescence was significantly decreased compared to miR-NC (*P* < 0.05). However, the relative luminescence increased when co-transfected with *circSVIL* expression vectors (Figure 2D). Both these two luciferase reporter assays suggested the negative effect of *circSVIL* expression on miR-203 activity.

In addition, RNA immunoprecipitation and biotin-coupled miRNA pull down in myoblast were also performed to validate the relation between *circSVIL* and miR-203. After co-transfected *circSVIL* with miR-203, miR-206, or miR-NC in myoblasts, immunoprecipitation of AGO2 was performed and the *circSVIL* expression levels were quantified by qRT–PCR and normalized to *GAPDH*. Compared to miR-NC or miR-206 transfected cells, immunoprecipitation of AGO2 from miR-203 transfected cells resulted in significant enrichment of the *circSVIL* (*P* < 0.01), suggesting that *circSVIL* could function as a binding platform for AGO2 and miR-203 (Figure 2E). Furthermore, compared to miR-NC or 3′-biotinylated miR-206 control, *circSVIL* was specifically captured by 3′-biotinylated miR-203 (*P* < 0.01) (Figure 2F). Altogether, these results suggested that a highly efficient interaction between *circSVIL* and miR-203.

### CircSVIL increased the expression of miR-203 target genes

**Figure 3:**
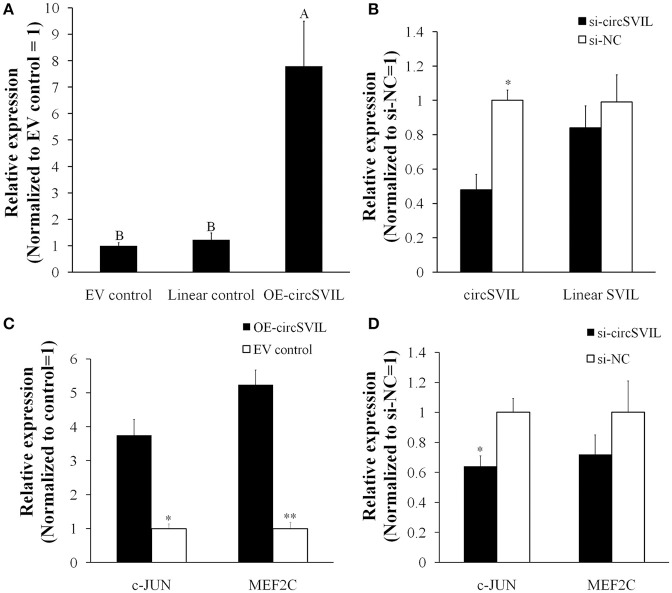
*CircSVIL* increased the expression of MEF2C and c-JUN, the target genes for miR-203. **(A)** Overexpression vector of *circSVIL i*ncreased the expression level of *circSVIL* but not linear *SVIL*. **(B)** siRNA of *circSVIL* decreased the expression level of *circSVIL* but not linear SVIL. **(C)** Overexpression of *circSVIL* increased the expression level of *MEF2C* and *c-JUN*. **(D)** Knockdown of *circSVIL* inhibited the expression level of *c-JUN*. The relative expression level was determined by qPCR 48 h after transfected with the overexpression vector (OE) or siRNAs of circRNAs. ^*^P < 0.05; ^**^P < 0.01; ^A,B^*P* < 0.01.

To observe the functions of *circSVIL* on chicken primary myoblast, we performed overexpression and knockdown of the *circSVIL* by transfected with overexpression vector or siRNAs. Green fluorescence images of primary myoblast and QM-7 transfected with *circSVIL* overexpression vector at 36 h were showed to evaluate the transfection efficiency (Figure S3). We also constructed the pcDNA3.1 vector with linear exon 6-14 of *SVIL* as control, which gives rise to linear *SVIL* products. After 48 h transfeced with overexpression vector in chicken primary myoblast, the expression level of *circSVIL* was efficient increased compared to empty vector or linear vector control (Figure 3A). Likewise, after 48 h transfection with siRNA of *circSVIL* in chicken primary myoblast, the qPCR results showed that the siRNAs could knockdown the *circSVIL* but not linear *SVIL* (Figure 3B).

The *c-JUN* and *MEF2C* are both direct targets of miR-203 in chickens, and their expression levels were inhibited by miR-203 (Luo et al., 2014). In this study, we determined their expression level after overexpression/knockdown of *circSVIL* in chicken primary myoblast. Compared to empty vector control, the expression of *c-JUN* and *MEF2C* was both increased (*P* < 0.05) by *circSVIL* overexpression vector (Figure 3C). Similarly, siRNA of *circSVIL* downregulated the expression of *c-JUN* gene compared to scrambled siRNA control (Figure 3D). The results that *circSVIL* upregulated the mRNA level of *c-JUN* and *MEF2C* also indicated that *circSVIL* could inhibit the activity of miR-203.

### CircSVIL promotes myoblast proliferation

**Figure 4:**
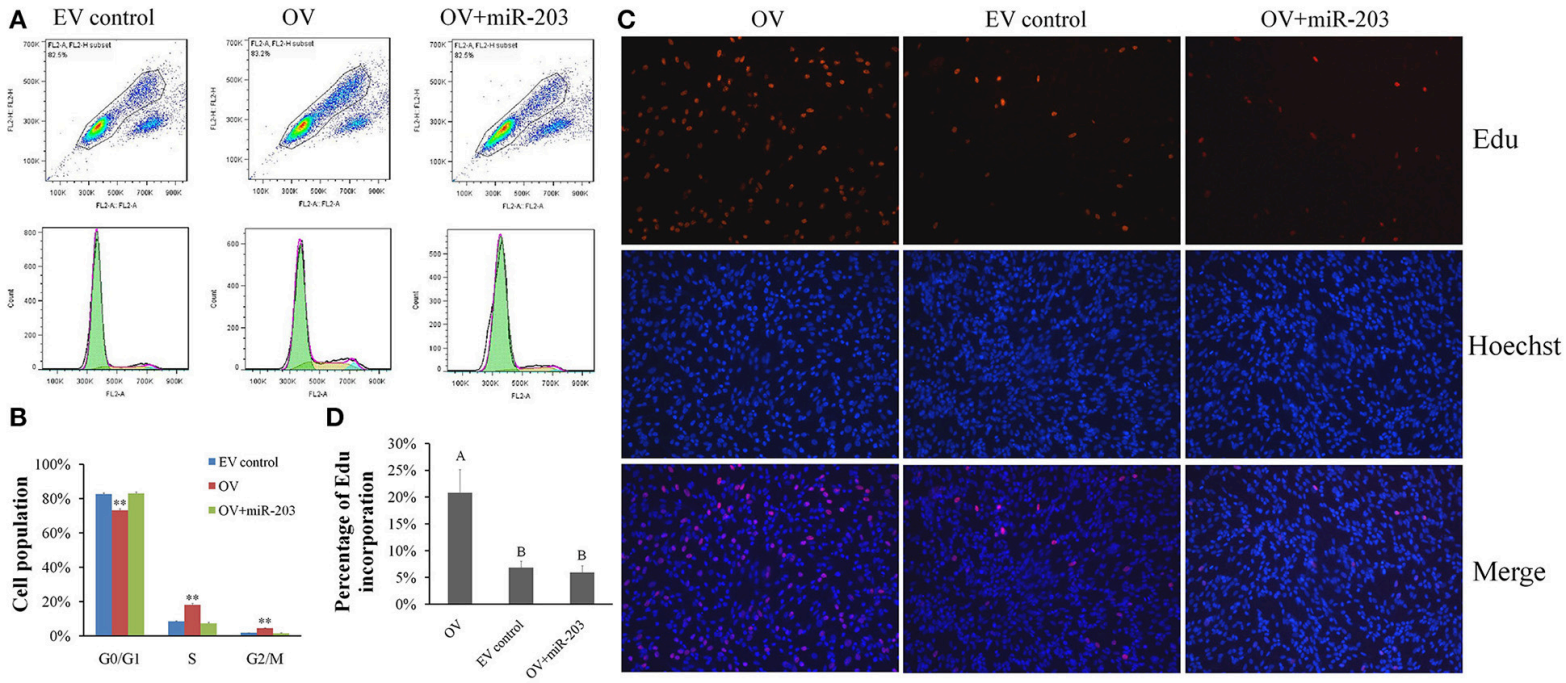
Chicken *circSVIL* promotes the proliferation of QM-7 cells. **(A)** Flow Cytometry raw data of cell cycle analysis for myoblast transfected with overexpression vector (OV) or empty vector control (EV) or co-transfected with overexpression vector and miR-203. **(B)** Overexpression of *circSVIL* increased the number of S and G2/M phase cells and decreased the number of G0/G1 cells. Bars represent S.D. (*n* = 4). **(C)** EdU assays for myoblast transfected with OV or EV or co-transfected with OV and miR-203. EdU (red) fluorescence indicates proliferation. Nuclei are indicated by Hoechst (blue) fluorescence. All photomicrographs are at 200 × magnification. **(D)** The percentage of EdU-stained cells per total cell numbers. Error bars represent S.D. (*n* = 3). Student's *t*-test (two-tailed) was performed for data analysis. ^*^P < 0.05; ^**^P < 0.01; ^a,b^*P* < 0.05; ^A,B^*P* < 0.01.

In chicken, miR-203 has been reported to inhibit the proliferation and differentiation of myoblast (Luo et al., 2014). Therefore, *circSVIL* also likely to play an important role on myoblast proliferation by acts as a sponge of miR-203. To observe the effects of *circSVIL* on myoblast proliferation, we performed flow cytometry for cell cycle analysis and 5-Ethynyl-2′-deoxyuridine (EdU) incorporation assays after 48 h overexpression or knockdown of the *circSVIL* in myoblast. Cell cycle analysis showed that overexpression of *circSVIL* resulted in a greater number of S and G2/M phase cells and fewer G0/G1 cells than control group (*P* < 0.01). However, when co-transfected with *circSVIL* and miR-203, the number of cells in each phase was no significantly different compared with the control group (Figures 4A,B). Similarly, compared to control group, the numbers of EdU-stained cells increased in overexpression group (*P* < 0.01) but not in co-transfected (*circSVIL* and miR-203) group (Figures 4C,D).

**Figure 5:**
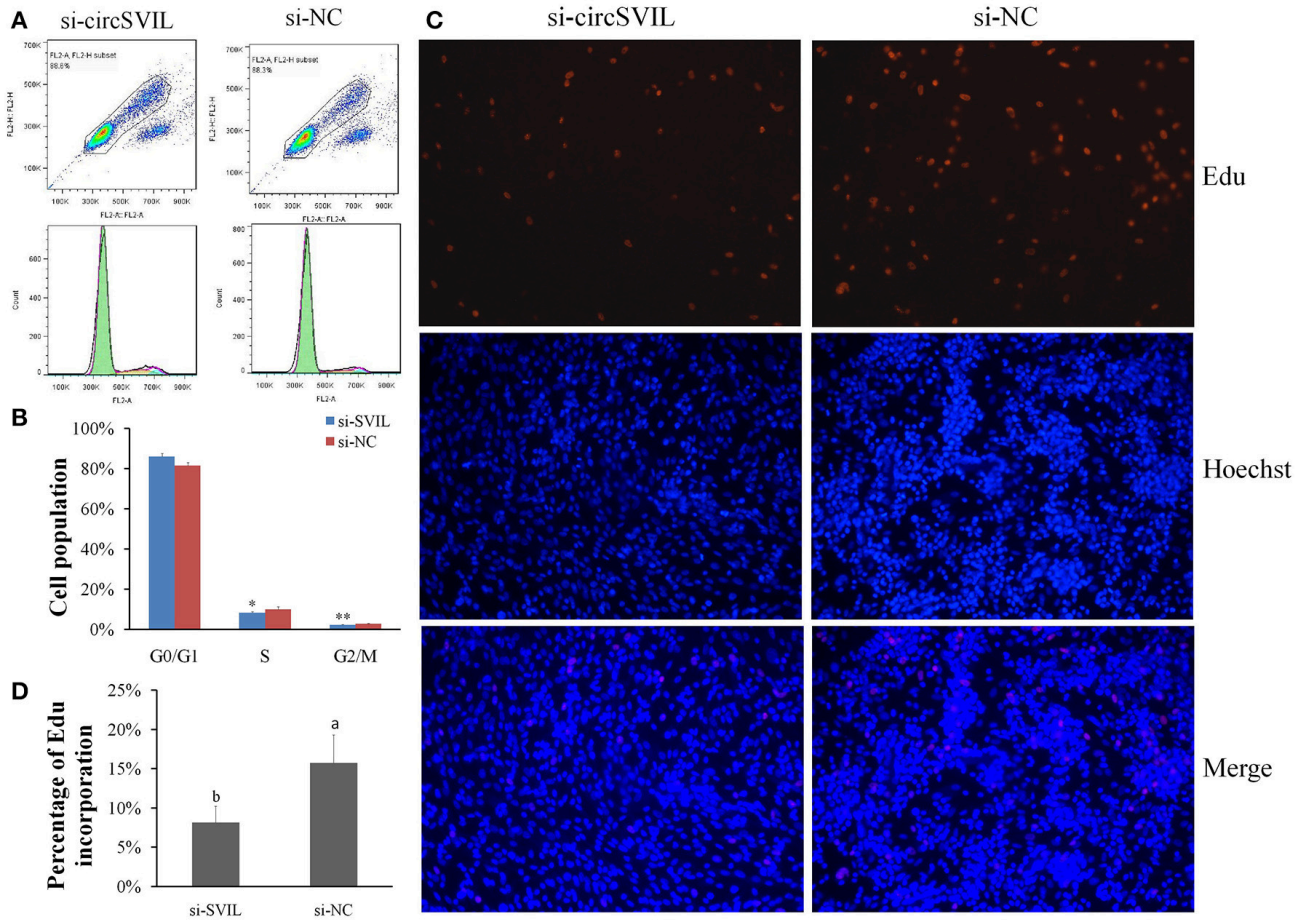
Knockdown of chicken *circSVIL* inhibits the proliferation of QM-7 cells. **(A)** Flow Cytometry raw data of cell cycle analysis for myoblast transfected with siRNA of *circSVIL* (si-*circSVIL*) or negative control (si-NC). **(B)** Knockdown of *circSVIL* decreased the number of S and G2/M phase cells. Bars represent S.D. (*n* = 4). **(C)** EdU assays for myoblast transfected with si-*circSVIL* or si-NC. EdU (red) fluorescence indicates proliferation. Nuclei are indicated by Hoechst (blue) fluorescence. All photomicrographs are at 200 × magnification. **(D)** The percentage of EdU-stained cells per total cell numbers. Error bars represent S.D. (*n* = 3). Student's *t*-test (two-tailed) was performed for data analysis. ^*^P < 0.05; ^**^P < 0.01; ^a,b^*P* < 0.05; ^A,B^*P* < 0.01.

After 48 h transfection of *circSVIL* siRNA in myoblast, we found that knockdown of *circSVIL* resulted in a fewer numbers of S (*P* < 0.05) and G2/M (*P* < 0.01) phase cells than si-NC group (Figures 5A,B). EdU incorporation assays results also showed that the percentage of EdU incorporation decreased when *circSVIL* was knocked down (Figures 5C,D). Altogether, these results suggested that *circSVIL* promotes myoblast proliferation, and impairs the activity of miR-203 in myoblasts.

### CircSVIL promotes myoblast differentiation

**Figure 6:**
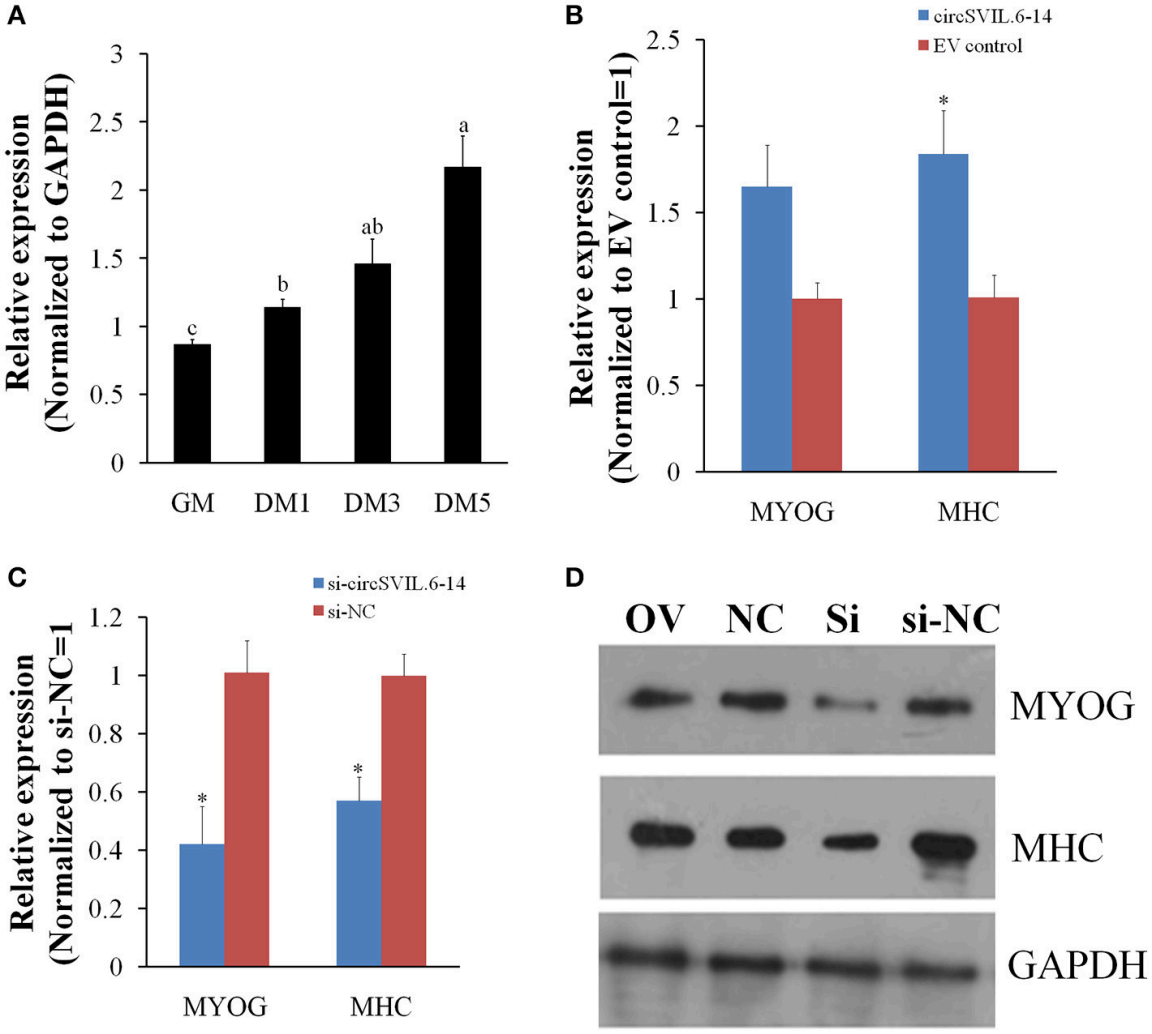
Chicken *circSVIL* promotes primary myoblast differentiation. **(A)** The expression of *circSVIL* was determined by qPCR during chicken primary myoblast differentiation. **(B)** The expression of MYOG and MHC was determined by qPCR in primary myoblast after transfected with *circSVIL*. **(C)** The expression of MYOG and MHC was determined by qPCR in primary myoblast after transfected with siRNA of *circSVIL*. **(D)** The expression of MYOG and MHC was determined by Western blot in primary myoblast after transfected with overexpression vector or siRNA of *circSVIL*.GM, cell with growth media; DM, cell with differential media. qRT-PCR reactions were run in triplicate and presented as means ± S.E.M. The Student's *t*-test was used to compare expression levels among different groups. ^*^P < 0.05; ^**^P < 0.011; ^a,b^*P* < 0.05.

To further study the potential role of *circSVIL* in myoblast differentiation, we detected the expression of *circSVIL* during the differentiation of myoblasts *in vitro*. The expression level of *circSVIL* in undifferentiated myoblasts with growth medium (GM) was lower than that in myoblasts with differentiation medium (DM), and gradually increased during the differentiation of myoblasts (Figure 6A). This result demonstrates that *circSVIL* expression is negative correlated with miR-203 levels, whose expression has been reported to downregulate from proliferation to differentiation of myoblasts (Luo et al., 2014).

Chicken primary myoblasts were cultured in GM to 100% confluency and then treated with DM to induce myoblast differentiation. After induced differentiation for 24 h, the cells were transfected with overexpression vector or siRNA of *circSVIL* and cultured in DM. Two major myoblast differentiation marker genes, *MYOG* (myogenin) and *MHC* (Myosin heavy chain) were detected after 48 h transfection using qRT-PCR and western blotting analysis (Chal and Pourquie, 2017).

The qRT-PCR results showed that overexpression of *circSVIL* increased the mRNA level of *MHC* (Figure 6B), while knockdown of *circSVIL* downregulated both the mRNA levels of *MYOG* and *MHC* (Figure 6C). As expected, the protein levels of *MYOG* and *MHC* were also upregulated by overexpression of *circSVIL* and downregulated by knockdown of *circSVIL* (Figure 6D). All these results indicate that *circSVIL* promotes myoblast differentiation *in vitro*.

## Discussion

In recent years, circRNAs have been identified widespread in various cell types or tissues of eukaryote and play an important role in many biological processes and human diseases. CircRNAs have been identified abundantly expressed in skeletal muscle of monkey and pig, and involving in growth and development of skeletal muscle (Abdelmohsen et al., 2015; Liang G. et al., 2017). Our previous study also found that circRNAs were abundant and dynamically expressed during embryonic muscle development in chicken by circRNA sequencing (Ouyang et al., 2017). In our circRNA sequencing data, *circSVIL* was the top abundant one in differentially expressed exonic circRNAs. In this study, we found that the expression of *circSVIL* was increased sharply from E11 to E14, and then maintained a high expression level during late embryo development. Functional circRNAs often show tissue and developmental stage specific expression patterns (Salzman et al., 2013; Westholm et al., 2014; Venø et al., 2015). The expression pattern of *circSVIL* indicates that it may promotes the development of skeletal muscle at late embryonic stage.

Since circRNA *CDR1as* was reported to affect brain function in zebrafish or mouse by sponging miR-7 (Hansen et al., 2013; Memczak et al., 2013; Piwecka et al., 2017), more and more circRNAs have been found to play various functions by serve as miRNAs sponge (Yamamura et al., 2017). *circ-ITCH* regulated the expression of *p21* and *PTEN* to inhibits bladder cancer progression by sponging miR-17/miR-224 (Yang et al., 2018). *circHIPK3* upregulates insulin secretion and beta cell activity by binds miR-124-3p and miR-338-3p to increase expression of *Slc2a2, Akt1* and *Mtpn* (Stoll et al., 2018). In addition, Studies have shown that artificially synthesized exogenous circRNAs can specifically inhibit the activity of miRNA *in vivo* (Jost et al., 2018). SVIL (supervillin) has F-actin binding activity, and promoting cell migration of F-actin-dependent (Brown et al., 2009; Fang et al., 2010). We found *circSVIL* was derived from exon 6 to 14 of *SVIL* (Gallus_gallus-4.0/galGal4) and harbored four potential binding sites for miR-203. Moreover, miR-203 was also reported to express differentially during chicken embryonic skeletal muscle development and to express highly at E12 and E14 (Luo et al., 2014). Therefore, we hypothesize that *circSVIL* could functions as miR-203 sponges in chicken. We validated this hypothesis by dual-luciferase reporter, RNA Immunoprecipitation and biotin-coupled miRNA pull down. The results that *circSVIL* upregulated the target genes of miR-203 also confirmed that *circSVIL* could sponging miR-203.

**Figure 7:**
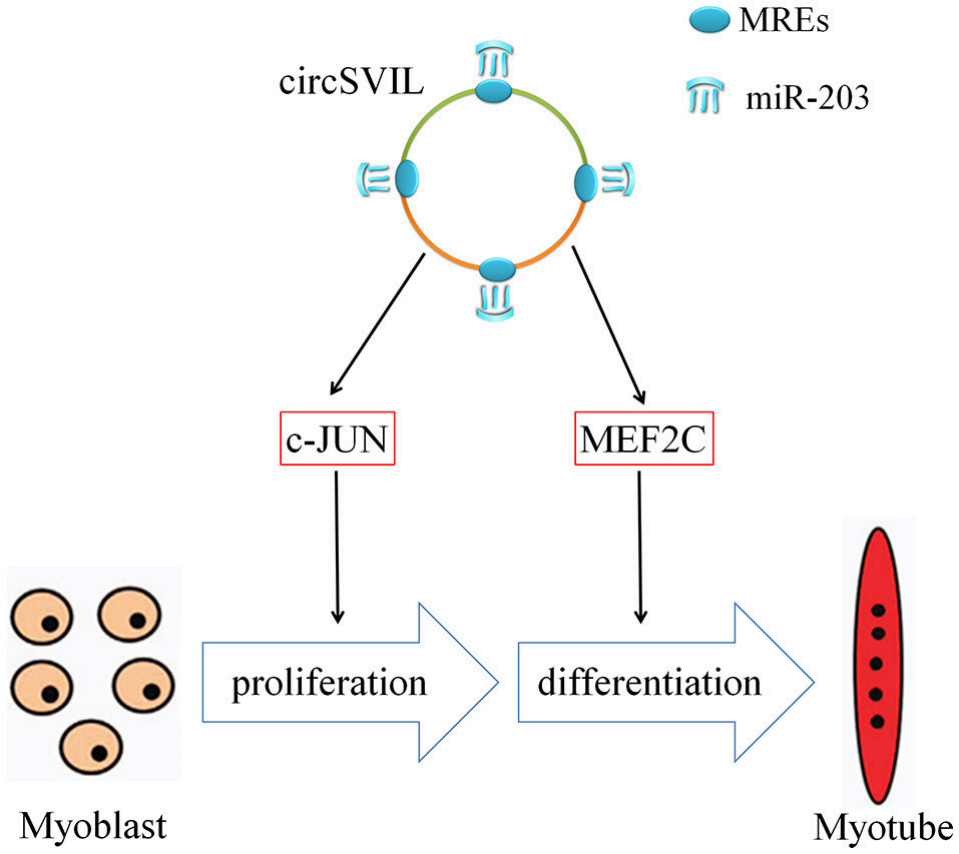
Mechanism of *circSVIL* in promoting skeletal muscle cell proliferation and differentiation through sponging miR-203. MRE, miRNA response element.

In mammalians, circRNAs can regulate muscle development by translated or act as sponge of miRNAs. Human *circ-ZNF609* was found that can be translated and functions in myogenesis (Legnini et al., 2017). The circRNA *circACTA2* increased the expression of smooth muscle *alpha-actin* through sponging miR-548f-5p (Sun et al., 2017). In this study, we also found that *circSVIL* was involved in chicken embryonic muscle development by sponging miR-203. The chicken miR-203 inhibits myoblast proliferation and differentiation (Luo et al., 2014). Through sponging miR-203, chicken *circSVIL* could inhibit the activity of miR-203 and thus promote skeletal muscle cell proliferation and differentiation (Figure 7).

## Conclusions

In summary, we found that chicken *circSVIL* could inhibit the activity of miR-203 and increased the expression of its genes *c-JUN* and *MEF2C* by sponging miR-203. Our results suggest that *circSVIL* could function as miR-203 sponge and therefore promote the embryonic skeletal muscle development.

## Author contributions

HO performed the experiments, analyzed the data, and wrote the manuscript; XC and ZL collected the samples and analyzed the data; WL performed the additional experiments and revised the manuscript; QN designed the study and reviewed the manuscript; XZ designed the study. All authors have read and approved the final manuscript.

### Conflict of interest statement

The authors declare that the research was conducted in the absence of any commercial or financial relationships that could be construed as a potential conflict of interest.

## Supplementary material

Figure S1 Experimental validation of *circSVIL*. **(A)** Divergent primers amplify *circSVIL* in cDNA but not genomic DNA (gDNA). White triangles represent convergent primers and black triangles represent divergent primers. **(B)** Sanger sequencing confirmed the back-splicing junction sequence of *circSVIL*. **(C)** qRT-PCR showing resistance of *circSVIL* to RNaseR digestion.

Figure S2 **(A)** Site 1 at position 393 nt; **(B)** Site 2 at position 1280 nt; **(C)** Site 3 at position 345 nt.

Figure S3 Green fluorescence images of primary myoblast **(A)** and QM-7 **(B)** transfected with circSVIL overexpression vector at 36 h.

Table S1 Detailed information of primers and RNA oligos. *F* represents the forward primer; *R* represents the reverse primer.
