# Supplementary figures and images for: Circular RNA circSVIL Promotes Myoblast Proliferation and Differentiation by Sponging miR-203 in Chicken
Source: Front Genet. 2018 May 16;9:172. doi: 10.3389/fgene.2018.00172 (PMC5964199; doi:10.3389/fgene.2018.00172)

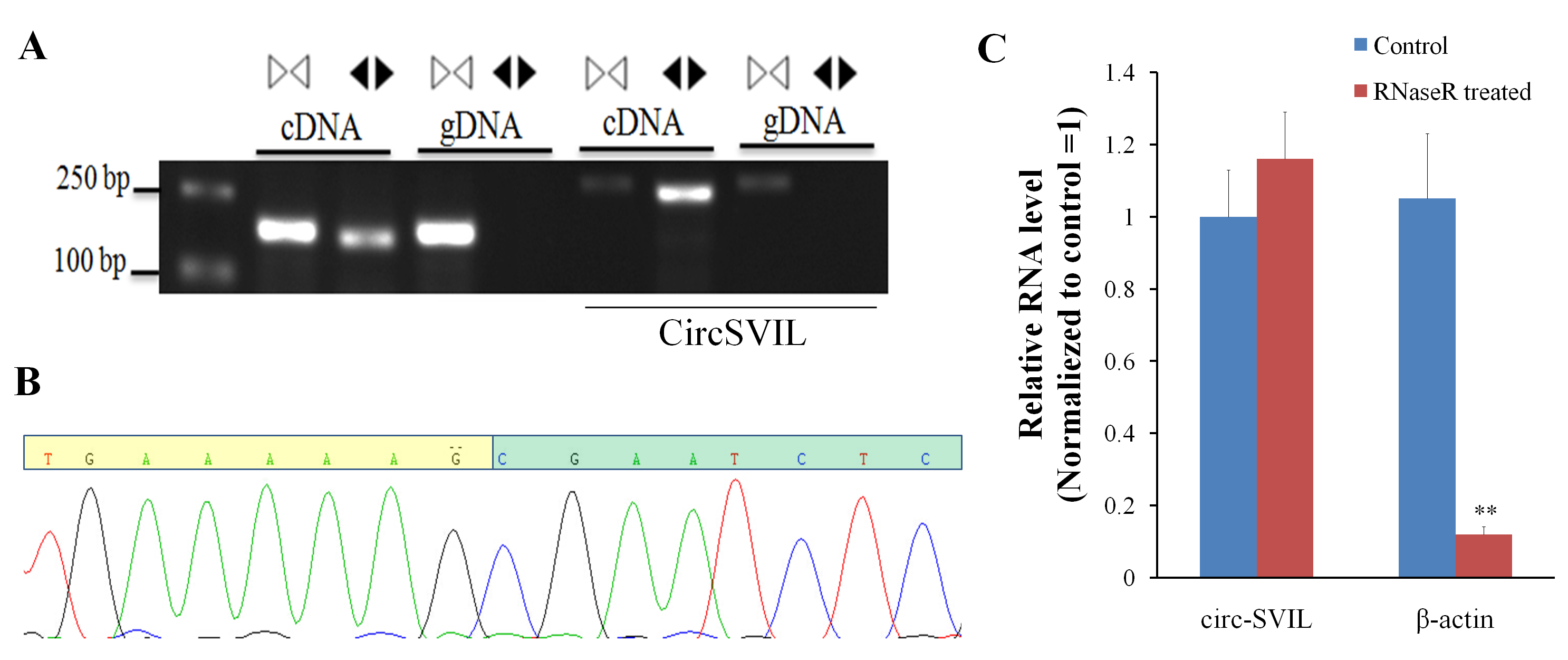

Supplement: Figure S1 — Experimental validation of circSVIL. (A) Divergent primers amplify circSVIL in cDNA but not genomic DNA (gDNA). White triangles represent convergent primers and black triangles represent divergent primers. (B) Sanger sequencing confirmed the back-splicing junction sequence of circSVIL. (C) qRT-PCR showing resistance of circSVIL to RNaseR digestion. [file Image_1.TIF]

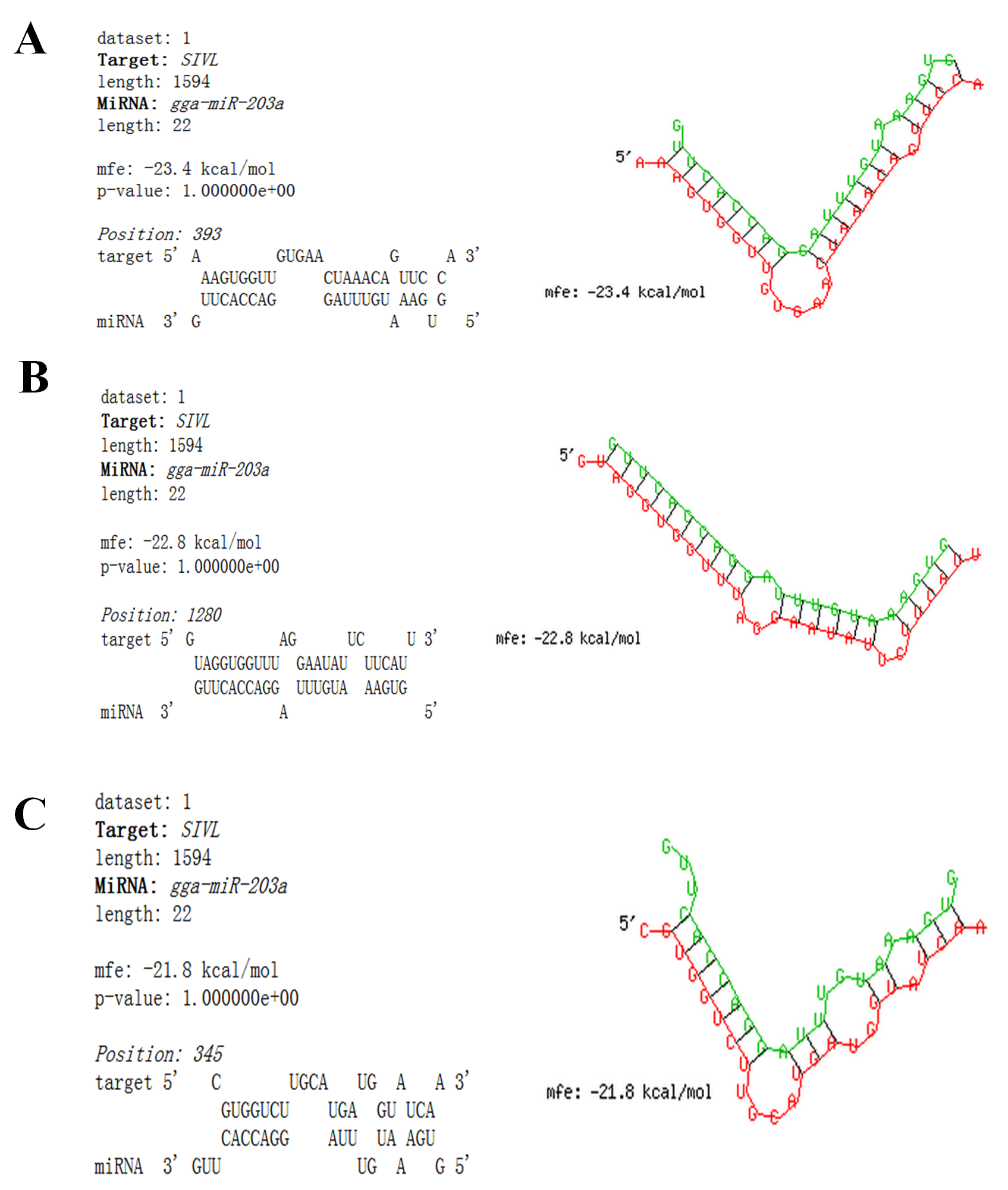

Supplement: Figure S2 — (A) Site 1 at position 393 nt; (B) Site 2 at position 1280 nt; (C) Site 3 at position 345 nt. [file Image_2.TIF]

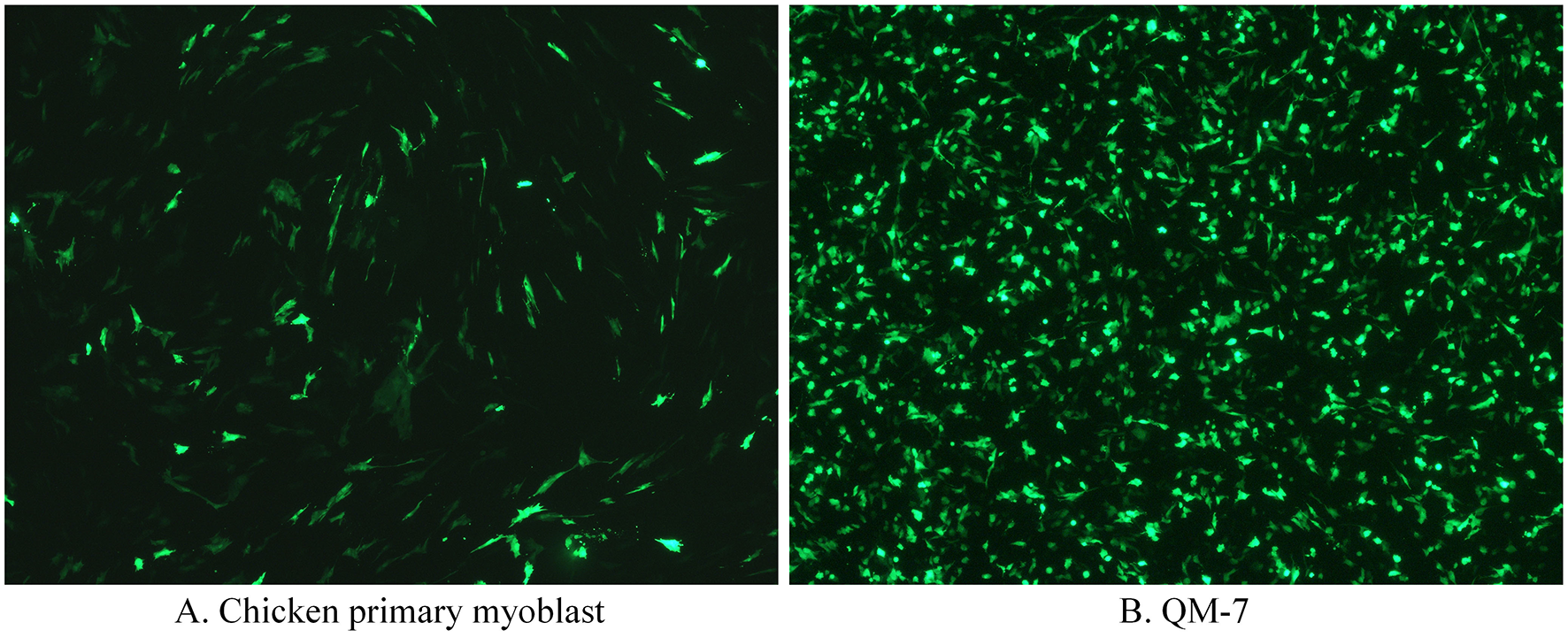

Supplement: Figure S3 — Green fluorescence images of primary myoblast (A) and QM-7 (B) transfected with circSVIL overexpression vector at 36 h. [file Image_3.tif]
