# Supplementary material for: Circular RNA circSVIL Promotes Myoblast Proliferation and Differentiation by Sponging miR-203 in Chicken
Source: Front Genet. 2018 May 16;9:172. doi: 10.3389/fgene.2018.00172 (PMC5964199; doi:10.3389/fgene.2018.00172)
Supplement: Table S1 — Detailed information of primers and RNA oligos. F represents the forward primer; R represents the reverse primer. [file Table_1.docx]

**Supporting Information**

**Table S1 Detailed information of primers and RNA oligos.**

| NO. | Nucleotide sequences (5’→3’） | Annealing temperature (℃) |
| --- | --- | --- |
| circSVIL  (divergent) | F: CACCAGGAGTGAAATAGCG | 60 |
|  | R: CAGCTGTTGAAAAAGCGAATC |  |
| circSVIL  (convergent) | F: AAAGGAGCTGTACTTTCTG  R: TGCACTGTTTGCTGGAGAC | 60 |
| MEF2C | F: ATCTCTCCCTGCCTTCTA | 60 |
|  | R: GTGATCTTCTCGGTCGCT |  |
| c-JUN | F: CCGCACCTGAGGAACAAGA | 58 |
|  | R: CCGTTGCTGGACTGGATGA |  |
| MYOG | F: CGGAGGCTGAAGAAGGTGAA | 60 |
|  | R: CGGTCCTCTGCCTGGTCAT |  |
| MHC | F: CTCCTCACGCTTTGGTAA | 58 |
|  | R: TGATAGTCGTATGGGTTGGT |  |
| GAPDH | F: CAGAACATCATCCCAGCGT | 56-64 |
|  | R: CAGGTCAGGTCAACAACAG |  |
| 18SrRNA | F: TGCATGTCTAAGTACACACGG | 58-62 |
|  | R: AGGTCGGCGCTCGTCGGCATG |  |
| siRNAs of circSVIL | AACCGAGAUUCGCUUUUUC | -- |

F represents the forward primer; R represents the reverse primer.
